# Reevaluating the rule of two's: Age and epidemiological variations in Meckel's diverticulum

**DOI:** 10.1002/jpn3.70521

**Published:** 2026-07-22

**Authors:** Ashley Polachek, Megha S. Mehta, Ahmed Shalaby, Aamirah Vadsariya, Wenjing Zong, David M. Troendle

**Affiliations:** ^1^ Division of Pediatric Gastroenterology UT Southwestern Medical Center Dallas Texas USA; ^2^ Children's Health Children's Medical Center Dallas Dallas Texas USA; ^3^ Lyda Hill Department of Bioinformatics UT Southwestern Medical Center Dallas Texas USA; ^4^ Informatics Coordinating Office University of Texas Southwestern Medical Center Dallas Texas USA

**Keywords:** congenital gastrointestinal anomalies, gastrointestinal hemorrhage, technetium Tc 99m pertechnetate scintigraphy, vitelline duct remnant

## Abstract

**Objective:**

To evaluate the clinical characteristics, diagnostic modalities, and age‐related variation of Meckel's diverticulum (MD) using data from a nationwide pediatric inpatient database and a single‐center institutional cohort.

**Methods:**

The Kids' Inpatient Database (KID) for 2016 and 2019 was queried for patients with a principal diagnosis of MD. We additionally conducted a retrospective review of patients hospitalized with a primary diagnosis of MD at our institution over a 20‐year period (January 1, 2005–May 1, 2025). Diagnosis was confirmed by surgical findings and pathology.

**Results:**

A total of 1004 patients with a primary diagnosis of MD were identified in KID. More than half were diagnosed after 5 years of age (*n* = 648, 64.8%). Similarly, among 147 patients identified in the single‐center cohort, 63.2% (*n* = 93) were diagnosed after 5 years of age. Within this single‐center cohort, patients ≤5 years more commonly presented with painless rectal bleeding (38.9%, *p* = 0.017) and underwent Meckel's scintigraphy more frequently (48.1%, *p* < 0.005). In contrast, patients >5 years more often presented with abdominal pain (61.3%) or vomiting (54.8%) and were more likely to undergo abdominal computed tomography (68.9%, *p* < 0.0001). Length of stay (LOS) was longer in patients <5 years and shortest in those >15 years (*p* = 0.04).

**Conclusion:**

More than half of pediatric patients with MD are diagnosed after 5 years of age. Presentation, LOS, and imaging patterns vary by age, with younger children more often presenting with bleeding and older children with obstructive symptoms and greater CT detection.

## Abstract (highlights)

**What is Known:**

- Meckel's diverticulum (MD) is traditionally considered a disease of early childhood and is commonly summarized by the “Rule of Twos”; presenting before the age of 2 years, found 2 ft. from the ileocecal valve, approximately 2 in. in length, and present in roughly 2% of the population.
- Classic presentation as described by the “Rule of Twos” is painless lower gastrointestinal bleeding.
- Technetium‐99m pertechnetate scintigraphy (Meckel's scan) is the preferred diagnostic test when MD is suspected.

**What is New:**

- More than half of pediatric patients with MD are diagnosed after 5 years of age.
- Clinical presentation varies significantly by age, with older children more often presenting with abdominal pain and vomiting while younger children with painless rectal bleeding.
- Computed tomography is more likely to be used to identify MD in older children and in obstruction‐predominant presentations, while bleeding‐predominant disease negatively predicted CT identification.

## 1 INTRODUCTION

Meckel's diverticulum (MD) is a common congenital anomaly with prevalence of 0.5%–3%, that occurs due to incomplete atrophy of the embryonic vitelline duct at the fifth week of gestation.^1^ Several studies have demonstrated a male predominance in MD, with males comprising up to 75% of cases.^1^, ^2^, ^3^ MD is classically a diagnosis of young childhood. Alemayehu et al.^3^ and Ruscher et al.^4^ found that more than half of all children diagnosed with MD who required surgical intervention were <5 years of age. Levy and Hobbs found 60% of patients presented with MD before 10 years of age.^5^ Additionally, consensus guidelines from the Society of Nuclear Medicine and Molecular Imaging and the European Association of Nuclear Medicine suggest that more than 50% of patients with MD who present with lower gastrointestinal bleeding do so by 2 years of age.^6^

The classic presentation of MD is painless lower gastrointestinal bleeding^7^, ^8^, ^9^ or obstructive symptomatology^1^, ^3^, ^10^; however, the symptoms of MD can vary widely including nausea, vomiting, pain, abdominal distension, intussusception, or even perforation. MD may also be found incidentally during surgical exploration. This poses a diagnostic challenge for the clinician.

Diagnostic modalities for MD include Meckel's scan (technetium‐99m nuclear medicine scan), capsule endoscopy, or computed tomography (CT). Less commonly, MD can be diagnosed by ultrasound (US) or magnetic resonance imaging. Capsule endoscopy is a non‐invasive technique that may also be utilized for identification of MD. This may be limited by patient's ability to swallow the capsule versus requiring endoscopic placement in the small bowel. A surgical approach including laparoscopy or laparotomy can be performed if there is high clinical suspicion or radiological evidence of MD. Ultimately, Meckel's diverticulectomy is the definitive treatment for those with symptomatic MD.

Histologic evaluation reveals heterotopic mucosa in up to 80% of cases. Most commonly gastric mucosa (24%–60%), followed by pancreatic tissue (5%–16%), with colonic reported rarely. Symptomatic MD is associated with a nearly tenfold higher incidence of heterotopic tissue compared with asymptomatic MD; however, only 50%–60% of patients with heterotopic tissue develop symptoms.^5^, ^11^

We hypothesized that MD is more common in older ages than previous dogma teaches. Given its historical association with infancy and atypical presentation in older children, we further posited that school‐age children and adolescents may experience a delayed diagnosis, as the condition may be lower on the differential in this population.

Our objective was to further investigate the clinical characteristics and the presentation of MD and diagnostic modalities utilized.

We collected data from a nationwide pediatric inpatient care database. The Healthcare Cost and Utilization Project Kid's Inpatient Database (HCUP KID) provides both clinical and financial information on pediatric hospitalizations. To achieve a more comprehensive understanding, we paired the population‐level data from HCUP KID with a focused institutional review that offered more detailed diagnostic and procedural insights.

## 2 METHODS

### 2.1 Ethics statement

This study was reviewed and approved by the Institutional Review Board of University of Texas Southwestern Medical Center. The requirement for informed consent was waived due to the retrospective nature of the study and use of de‐identified data.

### 2.2 Data sources and study population

The HCUP KID (https://www.hcup-us.ahrq.gov/overview.jsp) is the largest publicly accessible pediatric inpatient care database in the United States. It encompasses patient information from 44 states, providing national estimates of hospital stays for children defined as patients aged 20 years or younger at admission. HCUP KID's data are derived from administrative records and discharge summaries created by hospitals for billing purposes. The sampling rate in HCUP KID is 10% of uncomplicated in‐hospital births and 80% of all other pediatric discharges. The database is updated and released on a triennial basis.

For our study, we accessed the data for the years 2016 and 2019 which are the most recent years available. Data in HCUP KID were extracted using International Classification of Diseases, 10th edition, Clinical Modification (ICD‐10‐CM) and Procedure (ICD‐10‐PCS) codes. The database was queried for patients with a primary diagnosis of MD using ICD‐10 code of Q43.0. This information was stratified by age, diagnostic imaging, procedures, and length of hospital stay (in days) and analyzed.

A retrospective chart review was also conducted of pediatric patients hospitalized and diagnosed with MD at our institution between January 1, 2005, and May 1, 2025. Patients were identified through the electronic medical record system using International Classification of Diseases, 9th Revision (ICD‐9) and 10th Revision (ICD‐10) codes corresponding to MD as the primary diagnosis during hospitalization (751.0 and Q43.0, respectively).

### 2.3 Data collection

Inclusion criteria of our institutional retrospective review consisted of patients aged 0–20 years with a diagnosis of MD, through either surgical intervention or histopathological confirmation. No exclusion criteria were applied. Demographic and clinical data were extracted, including age at diagnosis, sex, presenting symptoms, diagnostic imaging modalities used, length of stay (LOS), and surgical outcomes.

Cases in both cohorts were similarly grouped: young children, ≤5 years of age; school age to early adolescence, 6–14 years of age; and mid to late adolescents, ≥15 years of age upon admission.

All data were de‐identified and collected in accordance with institutional review board guidelines. Descriptive statistics were used to summarize patient characteristics, and subgroup analyses were performed based on age ranges to identify patterns in clinical presentation and diagnostics.

### 2.4 Statistical analysis

Categorical variables, including race, ethnicity, presenting symptoms, diagnostic modalities, and ectopic tissue types, were compared across age groups using the chi‐square test of independence. Fisher's exact test was used when cell sizes were small (expected counts <5). Continuous and ordinal variables, such as age and LOS, were evaluated for normality. Group comparisons were performed using non‐parametric Kruskal–Wallis test for non‐normally distributed data including LOS. Additional chi‐square tests were conducted for race and ethnicity distributions within each age group to assess variation in demographic composition. A one‐sample proportion test was performed to determine whether White non‐Hispanic individuals were disproportionately represented in the cohort compared to an external reference population using an expected population proportion of 60%. Multivariable logistic regression models were used to evaluate how age and presenting symptoms influence the likelihood of MD detection on CT. All statistical tests were two‐sided unless otherwise specified, with a *p*‐value < 0.05 was considered statistically significant. Analyses were performed using R (version 4.2.2).

## 3 RESULTS

### 3.1 HCUP KID

A total of 1004 patients were identified with primary diagnosis ICD‐10 code for MD across 2016 (*n* = 513) and 2019 (*n* = 491) data sets. Racial and ethnic distribution remained relatively similar across both years (*p* = 0.609), with patients identifying as White/Caucasian comprising the majority (60.2% in 2016 vs. 57.2% in 2019), followed by Hispanic (19.9% vs. 20.2%) and African American (2.7% vs. 4.5%).

As seen in Table S1, age distribution was similar between the two cohorts without statistical difference; children aged 0–5 years accounting for the largest age proportion (35.7% in 2016 and 34.6% in 2019), but with the majority of patients being diagnosed after 5 years of age (64% in 2016 and 65% in 2019). The mean hospital LOS was comparable between age groups (4.84 ± 4.98 days in 2016 vs. 4.78 ± 4.87 days in 2019) [*p* = 0.341]). The distribution by hospital region appears to align with population distribution across U.S. regions. The patient cohort was almost evenly divided between with Medicaid coverage and those with private insurance which is comparable to the general population according to the 2023 American Academy of Pediatrics Policy Statement.^12^

### 3.2 Single‐center institution

#### 3.2.1 Demographics

A total of 147 patients diagnosed with MD were identified between January 1, 2005 and May 1, 2025 and analyzed across four age groups: ≤5 years (*n* = 54), 6–10 years (*n* = 30), 11–15 years (*n* = 33), and >15 years (*n* = 30). Males predominated across all age groups; however, the proportion of females was highest in the late adolescence group (40.0%), though this difference was not statistically significant. When comparing age distribution between HCUP KID and our single‐center institution we found no statistical significance (*p* = 0.56) (Figure 1). The racial distribution across all age groups was primarily those identified as White Non‐Hispanic. Median hospital LOS was highest among children 5 years and younger (5 days [interquartile range 4–6]) and declined with age which demonstrated statistical significance (*p* = 0.04) when comparing between all age cohorts (Table 1).

**Table 1:** Single center institution data: Patient demographics and admission details.

	Age ≤5 (*N* = 54)	Age 6–10 (*N* = 30)	Age 11–15 (*N* = 33)	Age >15 (*N* = 30)	*p*‐value
Female	14 (26.0%)	4 (13.3%)	6 (18.2%)	12 (40%)	0.0824
Mean age (years)	2.3	8.0	13.1	16.8	**‐**
Race					0.494
White	45 (83.3%)	22 (73.3%)	29 (87.9%)	24 (80%)	
African American	4 (7.4%)	1 (3.3%)	0 (0.0%)	1 (3.3%)	
Asian or Pacific Islander	0 (0.0%)	0 (0%)	0 (0%)	0 (0%)	
Native American	1 (1.9%)	1 (3.3%)	0 (0%)	0 (0%)	
Other	4 (7.4%)	6 (20.0%)	4 (12.1%)	5 (16.7%)	
Ethnicity					**0.0266**
Hispanic	25 (46.3%)	5 (15.3%)	8 (24.2%)	11 (36.7%)	
Non‐Hispanic	29 (53.7%)	25 (83.3%)	25 (75.8%)	19 (63.3%)	
Length of stay, median (interquartile range)	5 (4–6)	4 (3–6)	4 (2–6)	3 (2.3–4)	**0.0436**
Mean hemoglobin level	9.7	11.5	10.6	12.3	0.3916
Received packed red blood cell transfusion	20 (37.0%)	7 (23.3%)	17 (51.5%)	8 (26.7%)	0.0813

*Note*: Bold values indicate statistically significant at *p* < 0.05.

**Figure 1:**
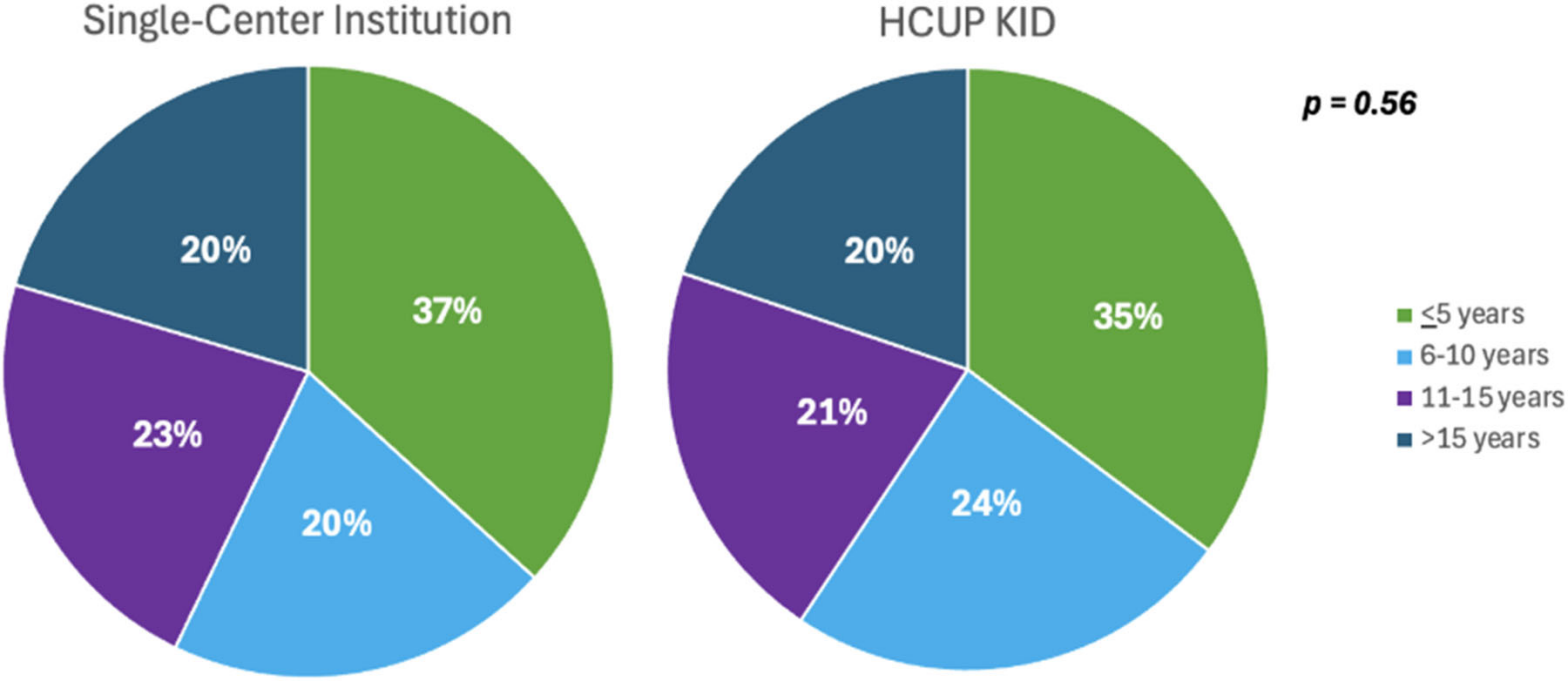
Age at diagnosis of Meckel's diverticulum between single‐center institutional data and HCUP KID. HCUP KID, Healthcare Cost and Utilization Project Kid's Inpatient Database.

#### 3.2.2 Presentation

Presenting symptoms varied significantly by age. Painless rectal bleeding was more common in patients ≤5 years (38.9%) and significantly decreased with age, falling to 10% in patients >15 years (*p* = 0.017). In contrast, abdominal pain without evidence of gastrointestinal (GI) bleeding was more common in older groups, observed in 73.3% of patients aged 6%–10% and 63.3% of those >15 (*p* ≤ 0.001). Vomiting was seen frequently throughout all age groups (43.3%–63.3%) (Table S2, Figure 2).

**Figure 2:**
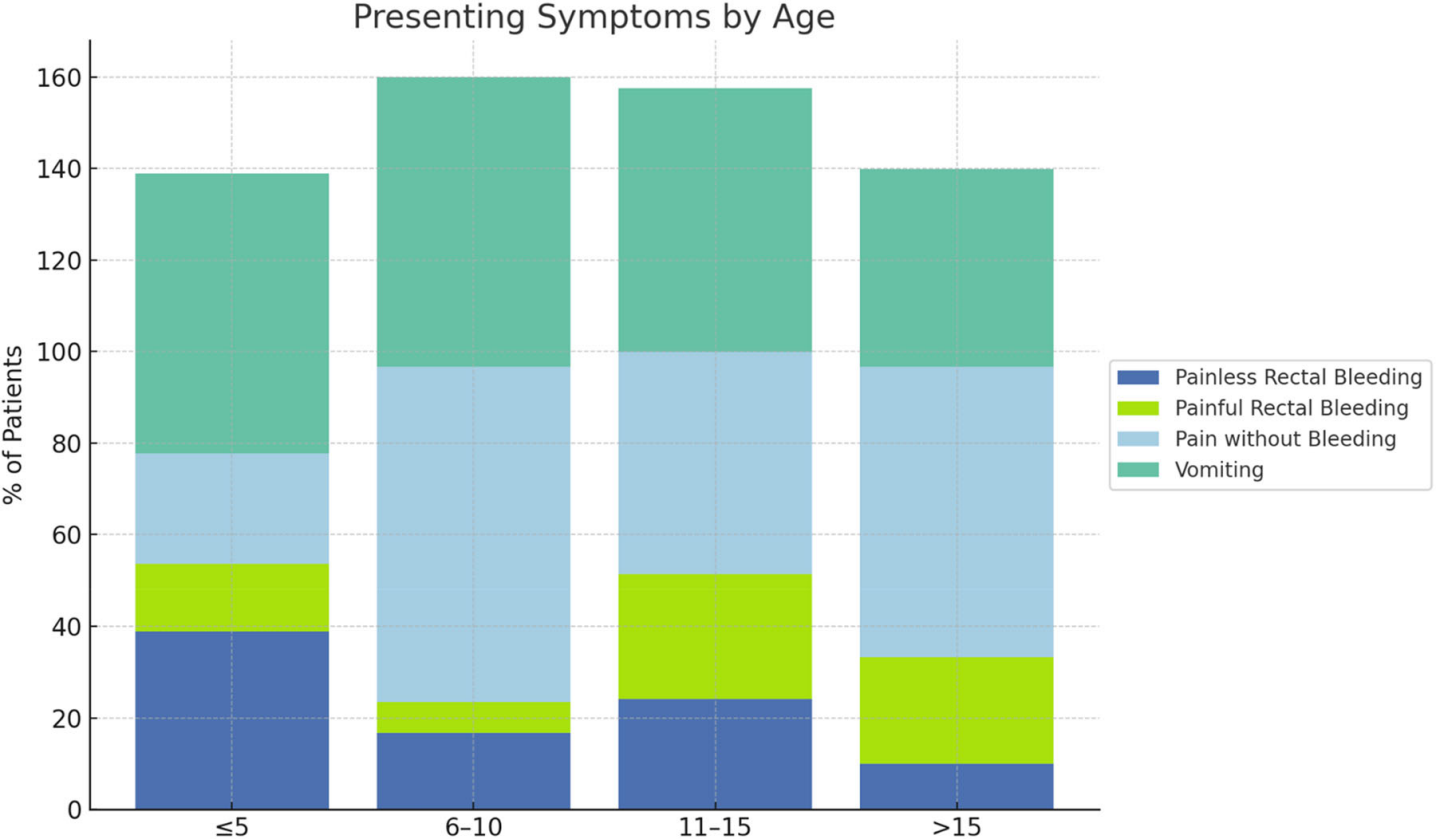
Presentation symptom(s) by age distribution at diagnosis of Meckel's diverticulum in single‐center institution data.

#### 3.2.3 Diagnostic evaluation

We identified several notable trends in the types of diagnostic studies performed in our patient population. Abdominal CT use increased with age, from 33.3% in children ≤5%–83.3% in those >15 (*p* < 0.001). Meckel's scan was most often obtained in the ≤5 age group (48.1%) with a high positivity rate (81%) (*p* = 0.005), while its utilization declined in older children (20%–45.5%) and adolescents (23.3%) (*p* < 0.005). US of the ileocecal region to assess for intussusception was performed in 64.8% of the youngest cohort but rarely in older groups (0%–6.7%) (*p* < 0.0001). Colonoscopy, while infrequently used, was more common in those >10, particularly ages 11–15 (24.2%) without statistical significance between age categories. Capsule endoscopy was rarely obtained (Table 2).

**Table 2:** Single‐center institution data: Diagnostic modalities and pathology.

	Age ≤ 5 (*N* = 54)	Age 6–10 (*N* = 30)	Age 11–15 (*N* = 33)	Age > 15 (*N* = 30)	*p*‐value
Diagnostics					
US Appendix	5 (9.3%)	12 (40.0%)	7 (21.2%)	4 (13.3%)	**0.0055**
US Ileocecal	35 (64.8%)	0 (0.0%)	1 (3.0%)	2 (6.7%)	**<0.0001**
Abdominal CT	18 (33.3%)	20 (66.7%)	19 (57.6%)	25 (83.3%)	**<0.0001**
Positive result?^a^	15 (83.3%)	14 (70.0%)	11 (57.9%)	15 (60.0%)	**0.3230**
Meckel's scan	26 (48.1%)	6 (20.0%)	18 (45.5%)	7 (23.3%)	**0.0048**
Positive result?	21 (81%)	2 (33.3%)	13 (72.2%)	6 (85.7%)	**0.0050**
H_2_RA given?	18 (69.2%)	4 (66.7%)	14 (77.8%)	4 (57.0%)	**0.0134**
Colonoscopy	6 (11.1%)	5 (16.7%)	8 (24.2%)	5 (16.7%)	0.4472
Positive result?^b^	0 (0.0%)	1 (20%)	0 (0.0%)	1 (20.0%)	0.2173
Capsule endoscopy	2 (3.7%)	2 (6.7%)	3 (9.1%)	2 (6.7%)	0.7298
Positive result?^c^	1 (50.0%)	0 (0.0%)	0 (0.0%)	1 (50.0%)	0.5051
Ectopic tissue					0.4946
Gastric	41 (75.9%)	17 (56.7%)	23 (69.7%)	20 (66.7%)	
Pancreatic	5 (9.3%)	5 (16.7%)	5 (15.1%)	2 (6.7%)	
None Identified	13 (24.1%)	13 (43.3%)	8 (24.2%)	9 (30%)	

*Note*: Bold values indicate statistically significant at *p* < 0.05.

Abbreviations: CT, computed tomography; H_2_RA, histamine‐2 receptor blocker; US, ultrasound.

^a^ Positive result of abdominal CT includes visualization of a blind‐tubular pouch, right lower quadrant mass, small bowel obstruction, enterolith, or intussusception.

^b^ Positive result of colonoscopy includes visualization of ileal lesion/mass/ulcer or active bleeding.

^c^ Positive result of capsule endoscopy includes visualization of diverticulum entrance or ileal lesion/mass/ulcer.

Multivariable logistic regression models were utilized to evaluate how age and presenting symptoms may influence the likelihood of MD detection on CT. CT detection of MD was less likely in bleeding‐predominant presentations; while vomiting and obstruction‐related symptoms were associated with higher odds of CT positivity, especially in older children (Figures S1 and S2). These findings suggest that CT may be more useful when evaluating older patients with obstructive symptomatology rather than those with painless GI bleeding.

Gastric ectopic tissue was the most common histopathologic finding identified across all age groups. Pancreatic tissue was less common overall but more frequently seen in ages 6–15 years, which would limit the utility of Meckel's scan in this group. No statistically significant difference in type of heterotopic tissue was observed between age cohorts.

## 4 DISCUSSION

Classic dogma of the “Rule of 2's” suggests MD primarily affects young children under 5 years of age; however, our findings challenge this assumption with nearly two‐thirds presenting >5 years old. This expanded age distribution highlights the need for sustained clinical vigilance across a broader pediatric age range, ensuring that MD remains a diagnostic consideration beyond early childhood.

Several factors may lead to the presentation of MD later in life. MD may remain asymptomatic until complications like bleeding or obstruction occur. Increased physical activity in older children may contribute to mechanical manipulation, however speculative while the gradual development of ulceration from ectopic gastric mucosa may play a role. Additionally, improved symptom articulation in older patients may facilitate more accurate identification.

Symptomatology also varied significantly with age. In children ≤5 years, classic painless rectal bleeding was the most common presentation. However, as age increased, patients more frequently presented with abdominal pain without gastrointestinal bleeding, often accompanied by vomiting. Prior dogma and presenting symptoms not classically attributed to MD may delay diagnosis in older populations as other abdominal pathologies such as appendicitis or inflammatory bowel disease are being more highly considered and evaluated for.

Diagnostic imaging trends further reflected age‐related practice variation. Meckel's scan was most commonly utilized in the youngest cohort, with a high rate of diagnostic yield, while abdominal CT was increasingly obtained in older patients with a higher rate of diagnostic yield in comparison to younger children. Based off prior radiologic studies, we reported a positive result on CT as visualization of a blind‐tubular pouch, right lower quadrant mass, small bowel obstruction, enterolith or intussusception.^13^, ^14^ These imaging findings were further confirmed to represent MD based on diagnostic codes and surgical pathology.

Although without statistical significance, school age children and early adolescents were found to have a higher prevalence of pancreatic tissue which will not be identified on Meckel's scan. This likely contributes to the lower clinical suspicion and potential increased reliance on cross‐sectional imaging in older pediatric populations. These findings raise the question of whether greater consideration should be given to cross‐sectional imaging, such as CT, in younger children, particularly since MD was detectable on CT in more than 50% of patients in all age groups. With multivariable logistic regression analysis, both vomiting and increasing age were associated with higher odds of CT identification of MD, while bleeding‐predominant presentations and abdominal pain were associated with lower odds of detection. Vomiting likely reflects mechanical complications of MD, including obstruction or intussusception, which produce gross anatomic changes more readily detected on CT. We found that older children more commonly present pain likely from these mechanical‐type complications, accounting for their higher likelihood of CT detection. In contrast, bleeding‐predominant disease reflects a mucosal process without gross structural change and is therefore less likely to be visualized on CT. These findings suggest that CT may be more useful when evaluating older patients with obstructive symptomatology rather than those with painless GI bleeding.

Contrary to our hypothesis, older children did not experience longer hospital stays. This may be driven by earlier CT‐based diagnosis in the emergency department, whereas Meckel's scan requires nuclear medicine availability and is often delayed until inpatient admission.

By combining a large, nationally representative dataset with a detailed single‐center review, we were able to provide representative data with consistent patterns in age at diagnosis. Together, these complementary datasets strengthen the validity and clinical relevance of our findings.

Our study has limitations, including retrospective design, introducing potential selection bias and limiting control of confounding variables. HCUP KID relied on administrative coding, which may be incomplete, inaccurate, or lack detailed clinical information. Additionally, the institutional data spanned two decades, during which changes in diagnostic technology and clinical practice may have influenced observed trends.

Further research is warranted to refine diagnostic pathways for MD, as current imaging approaches, even when guided by age and clinical presentation may still miss a number of cases, with potential implications for delayed diagnosis and surgical management.

## 5 CONCLUSION

The prior dogma of the longstanding “Rule of Twos” for MD is outdated. Our findings emphasize the need for clinicians to broaden their diagnostic consideration of MD across all pediatric age groups through young adulthood. Overreliance on age‐based assumptions and presenting symptoms associated with this paradigm may lead to missed or delayed diagnoses, especially in patients who present with atypical features.

## CONFLICT OF INTEREST STATEMENT

The authors declare no conflicts of interest.

## Supporting information


**Supplemental Figure 1**. Forest plot of variables in CT detection of Meckel's Diverticulum in Single Center Institution Data showing odds ratios and 95% confidence intervals for each predictor/symptoms across age groups.

**Supplemental Figure 2**. Predicted probability of Meckel's Diverticulum detection on CT versus age from multivariable model in Single‐Center Institution Data.

**Supplemental Table 1**. HCUP KID ‐ Patient Demographics and Hospital Characteristics.

**Supplemental Table 2**. Presenting Symptoms by Age Stratification.
