# Supplementary material for: Reevaluating the rule of two's: Age and epidemiological variations in Meckel's diverticulum
Source: J Pediatr Gastroenterol Nutr. 2026 Jul 22;83(4):658–64. doi: 10.1002/jpn3.70521 (PMC13646026; doi:10.1002/jpn3.70521)
Supplement: Supplementary file 1 [file JPN3-83-658-s001.docx]

**Presenting Symptom(s)**	**Age ≤5** *(N=54)*	**Age 6-10** *(N=30)*	**Age 11-15** *(N=33)*	**Age >15** *(N=30)*	**P-Value**
Painless rectal bleeding	21 (38.9%)	5 (16.7%)	8 (24.2%)	3 (10%)	**0.0167**
Painful rectal bleeding	8 (14.8%)	2 (6.7%)	9 (27.2%)	7 (23.3%)	0.1358
Pain without bleeding	13 (24.1%)	22 (73.3%)	16 (48.5%)	19 (63.3%)	**<0.0001**
Vomiting	33 (61.1%)	19 (63.3%)	19 (57.6%)	13 (43.3%)	0.3683
Fever	8 (14.8%)	3 (10%)	1 (3.0%)	2 (6.7%)	0.3218
