# Supplementary material for: Reevaluating the rule of two's: Age and epidemiological variations in Meckel's diverticulum
Source: J Pediatr Gastroenterol Nutr. 2026 Jul 22;83(4):658–64. doi: 10.1002/jpn3.70521 (PMC13646026; doi:10.1002/jpn3.70521)
Supplement: Supplementary file 2 [file JPN3-83-658-s002.docx]

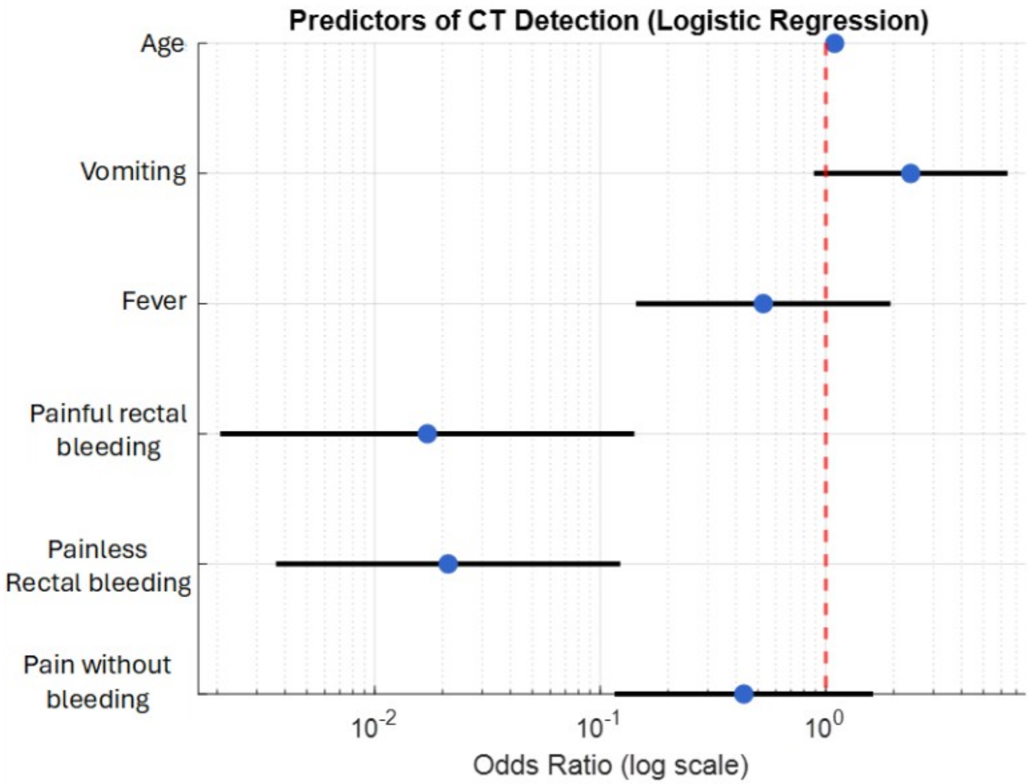
Predictors of CT Detection (Logistic Regression)
Age
Vomiting
Fever
Painful rectal bleeding
Painless Rectal bleeding
Pain without bleeding
10^{-2}
10^{-1}
10^{0}
Odds Ratio (log scale)
