# Supplementary material for: Reevaluating the rule of two's: Age and epidemiological variations in Meckel's diverticulum
Source: J Pediatr Gastroenterol Nutr. 2026 Jul 22;83(4):658–64. doi: 10.1002/jpn3.70521 (PMC13646026; doi:10.1002/jpn3.70521)
Supplement: Supplementary file 3 [file JPN3-83-658-s003.docx]

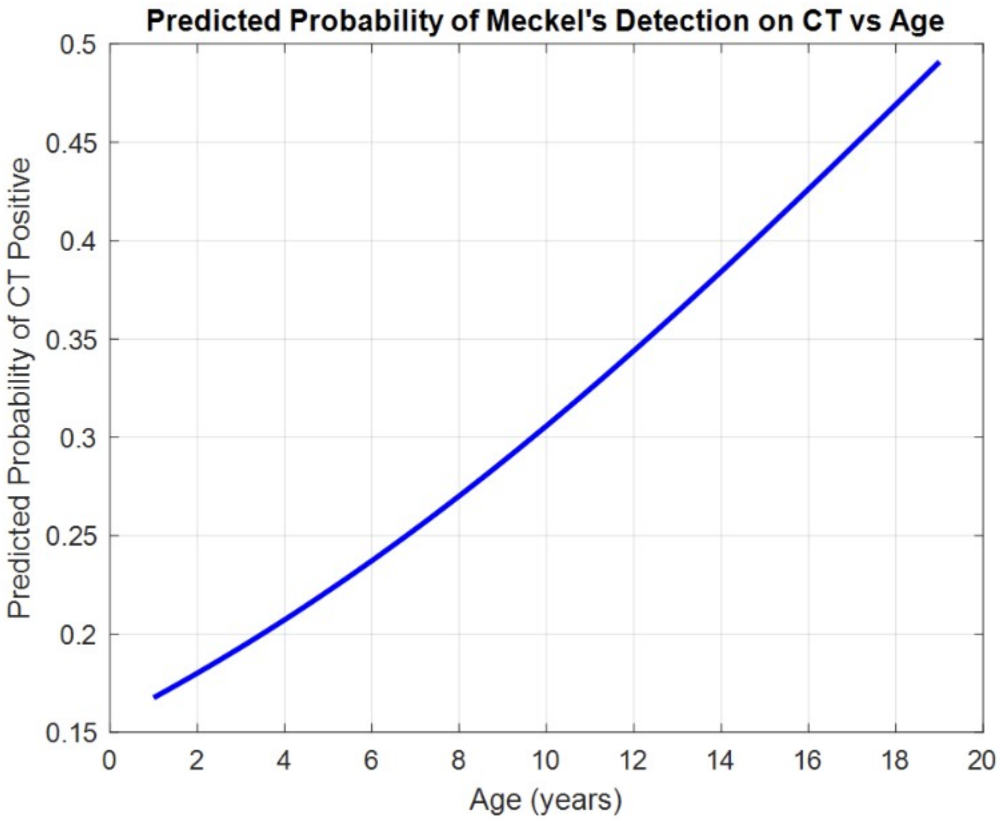
Predicted Probability of Meckel's Detection on CT vs Age
Predicted Probability of CT Positive
0.5
0.45
0.4
0.35
0.3
0.25
0.2
0.15
0
2
4
6
8
10
12
14
16
18
20
Age (years)
