# Supplementary material for: Reevaluating the rule of two's: Age and epidemiological variations in Meckel's diverticulum
Source: J Pediatr Gastroenterol Nutr. 2026 Jul 22;83(4):658–64. doi: 10.1002/jpn3.70521 (PMC13646026; doi:10.1002/jpn3.70521)
Supplement: Supplementary file 4 [file JPN3-83-658-s004.docx]

	KID 2016 (n=513)	KID 2019 (n=491)	P-Value
Female	116 (22.6%)	126 (25.7%)	0.291
Race			0.609
White	309 (60.2%)	291 (57.2%)	
African American	14 (2.7%)	22 (4.5%)	
Hispanic	102 (19.9%)	99 (20.2%)	
Asian or Pacific Islander	25 (4.9%)	29 (5.9%)	
Native American	4 (0.8%)	5 (1.0%)	
Age *(years)*			0.598
0-5	183 (35.7%)	170 (34.6%)	
6-10	121 (23.6%)	121 (24.6%)	
11-15	100 (19.5%)	109 (22.2%)	
16-20	108 (21.1%)	91 (18.5%)	
Duration of stay (*days*), mean (*SD*)	4.84 (+/- 4.98)	4.78 (+/- 4.87)	0.341
Region of Hospital			0.353
Northwest	76 (14.8%)	77 (15.7%)	
Midwest	128 (25.0%)	109 (22.2%)	
South	162 (31.6%)	178 (36.3%)	
West	147 (28.7%)	127 (25.9%)	
Insurance			0.103
Medicare	2 (0.4%)	0 (0.0%)	
Medicaid	186 (36.3%)	210 (42.8%)	
Private	282 (55.0%)	242 (49.3%)	
Other	40 (7.8%)	37 (7.5%)	
